# Clinicopathological Analysis of Soft Tissue Tumors and Tumor-Like Lesions of the Fingers in an Industrial Region: A Retrospective Evaluation of 134 Patients

**DOI:** 10.7759/cureus.99801

**Published:** 2025-12-21

**Authors:** Ümit Gök, Veysel Emre Çelik, Nazli Demir Gök, Baki Avşar Uzun

**Affiliations:** 1 Orthopedics and Traumatology, University of Health Sciences, Kocaeli City Hospital, İzmit, TUR; 2 Pathology, University of Health Sciences, Kocaeli City Hospital, İzmit, TUR; 3 Hand Surgery, University of Health Sciences, Kocaeli City Hospital, İzmit, TUR

**Keywords:** finger, ganglion cyst excision, soft tissue tumor, tendon sheath, tumor

## Abstract

Aim

This study aimed to determine the clinicopathological spectrum and incidence of soft tissue tumors and tumor-like lesions of the fingers in Kocaeli and to evaluate whether occupational factors in a highly industrialized region influence their distribution.

Methods

Of 218 patients who underwent surgery for soft tissue masses of the hand and wrist between December 2014 and July 2025, 134 patients (61.4%) with masses located in the fingers were included. Patient demographics, lesion side (right/left), lesion location (dorsal/volar), clinical pre-diagnosis, and pathological diagnosis were analyzed. A p-value <0.05 was considered statistically significant.

Results

The mean age of patients was 47.6 ± 13.5 years, and 50.7% were male. Lesions were located on the right hand in 48.5% of cases, with the most frequently affected fingers being the third (32.8%) and first (25.4%) fingers. The most common tumor was the tendon sheath giant cell tumor (TSGCT) (37.3%), followed by the ganglion cyst (14.2%). Epidermal cysts were significantly more common in men (p = 0.039). The overall concordance between clinical and pathological diagnoses was 76.1%, with fibroma being the most frequently misdiagnosed lesion.

Conclusions

All excised soft tissue tumors and tumor-like lesions of the fingers were benign. TSGCT was the most common tumor in this series, with a higher prevalence than previously reported in the literature. Clinical diagnoses are generally reliable, although rare lesions can be challenging. Because complete excision is particularly important for TSGCT and glomus tumors, combining clinical, radiological, and histopathological evaluation of finger masses can help ensure accurate diagnosis and appropriate treatment.

## Introduction

Approximately 15% of soft tissue masses occur in the fingers [1]. Tumors that can arise anywhere in the musculoskeletal system may also be seen in the fingers, spanning a wide spectrum from non-neoplastic conditions to malignant tumors [2]. However, the vast majority are benign [3]. Ganglion cysts (GCs) and tendon sheath giant cell tumors (TSGCTs) account for 50-70% of benign soft tissue tumors [4].

The primary concerns associated with finger masses include cosmetic appearance, limited movement, and fear of malignancy, as these masses are often painful [5]. In most cases of finger tumors operated on by experienced surgeons, the initial diagnosis based on clinical examination and radiological imaging correlates well with the histopathological findings [6]. Total excisional biopsy is generally the treatment of choice for these masses [7].

Although the literature does not definitively establish a link between chronic trauma and hand tumors, we hypothesize that in regions with a high concentration of industrial workers, factors such as chronic trauma and chemical exposure may influence the type and frequency of hand tumors.

The aim of this study was to determine the incidence of soft tissue tumors in patients presenting with finger masses in Kocaeli, which ranks first in Turkey in terms of industrial employment relative to its population and is among the top five cities in industrialization and industrial-sector employment. The study also aimed to retrospectively evaluate the clinical and pathological features of these tumors in light of the existing literature.

## Materials and methods

Study design and ethical approval

This retrospective study examined 218 patients who underwent surgery for soft tissue masses of the hand and wrist at Kocaeli City Hospital and Kocaeli Izmit SEKA State Hospital Orthopedics and Traumatology Clinics between December 2014 and July 2025. Of these, 134 patients (61.4%) had masses located in the fingers. The study was approved by the Kocaeli City Hospital Scientific Research Ethics Committee on November 13, 2025, with retrospective study protocol code 2025-138. All procedures were conducted in accordance with the ethical principles outlined in the Declaration of Helsinki.

Patient selection

**Figure 1:**
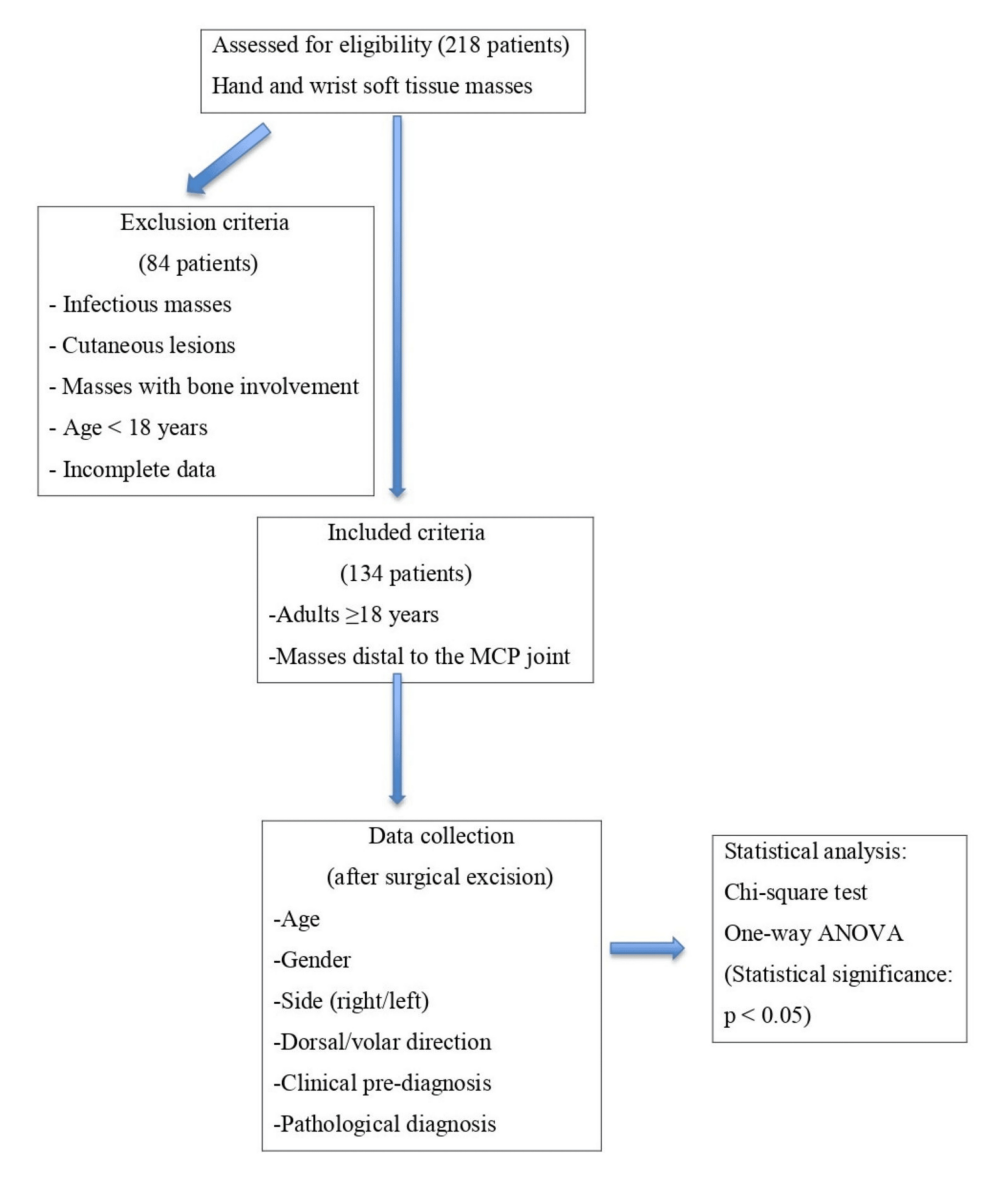
Study flowchart MCP, metacarpophalangeal

Finger localization was defined from the base of the metacarpophalangeal joint to the fingertip. Patient demographics (age, gender), lesion side (right/left hand), lesion location on the finger (dorsal/volar), clinical pre-diagnosis, and pathological diagnoses were obtained from patient files and electronic medical records (Figure 1).

Inclusion and exclusion criteria

Patients included in the study were over 18 years of age, had masses located distal to the metacarpophalangeal joint (first to fifth fingers), and had complete clinical, demographic, and pathological data available in their medical records. Patients were excluded if they had infectious masses, cutaneous lesions, or masses with bone involvement identified during histopathological evaluation.

Preoperative evaluation

Preoperative radiographs and ultrasonography were obtained for all patients, and an MRI was performed when recommended by the radiologist. Imaging-guided preoperative surgical planning. MRI examinations followed standard hand/wrist protocols, including T1-weighted, T2-weighted, and fat-suppressed sequences in axial and coronal planes to characterize lesions and plan surgery.

Surgical procedure

Patients were admitted on the morning of surgery. Those at risk of infection received 1 g of intravenous cefazolin sodium one hour before tourniquet application. The type of anesthesia was selected based on lesion location, patient age, and surgical technique. Local anesthesia was administered using 10 mL of 2% prilocaine hydrochloride, while patients unable to tolerate local anesthesia underwent general anesthesia. Masses were excised as completely as possible, including marginal tissue. If necessary, a finger tourniquet or an arm tourniquet at 250 mmHg was applied for hemostasis. Intraoperative pathology consultation was not performed.

Clinical preliminary diagnoses were made preoperatively by orthopedic surgeons specializing in hand surgery. Excised specimens were sent for postoperative pathological examination, and the surgeon’s preliminary diagnosis was reported to the pathologist. Histopathological diagnoses were made by experienced musculoskeletal pathologists according to standard WHO classification criteria. Results were recorded during follow-up visits.

Postoperative care

Antibiotic prophylaxis continued for three days postoperatively. Oral nonsteroidal anti-inflammatory drugs were administered for analgesia. Patients not requiring immobilization were advised to perform finger exercises immediately after surgery and were discharged the same day. Dressings were recommended every three days post-discharge, and sutures were removed between days 12 and 15. Patients with limited joint movement were referred to physical therapy and rehabilitation.

Statistical analysis

Statistical analysis was performed using IBM SPSS Statistics for Windows, Version 25.0 (Released 2017; IBM Corp., Armonk, NY, USA). Numerical variables were presented as mean ± SD, median (25th-75th percentiles), or frequency (percentages). Relationships between pathological and preliminary diagnoses, age groups, gender, lesion side (right/left), and location direction (dorsal/volar) were evaluated using the chi-square test. One-way ANOVA was used to compare continuous variables (age) between different tumor types, according to the normality of distribution. Statistical significance was set at p < 0.05.

## Results

A total of 134 patients were included in the study. The mean age was 47.6 ± 13.5 years. Of these, 68 patients (50.7%) were male, and 66 (49.3%) were female. Lesion distribution was 65 (48.5%) on the right hand and 69 (51.5%) on the left hand. Regarding lesion location, 60 patients (44.8%) had dorsal lesions, while 74 patients (55.2%) had volar lesions. Patient demographics are summarized in Table 1.

**Table 1:** Demographic data of all tumors and tumor-like lesions Demographic data are presented as n (%) and mean ± SD.

Variable	Value
Total patients	134
Age (years)	47.6 ± 15.3
Sex, n (%)	
Male	68 (50.7%)
Female	66 (49.3%)
Side, n (%)	
Right	65 (48.5%)
Left	69 (51.5%)
Direction, n (%)	
Dorsal	60 (44.8%)
Volar	74 (55.2%)
Finger, n (%)	
1	34 (25.4%)
2	26 (19.4%)
3	44 (32.8%)
4	16 (11.9%)
5	14 (10.4%)

No significant difference was observed between the dorsal and volar distribution of finger tumors (p = 0.111). However, all glomus tumors were located on the dorsal side, which was statistically significant (p = 0.027). Most TSGCTs and epidermal cysts were observed on the volar side, while GCs showed a balanced distribution between dorsal and volar regions. No significant difference was found between the right and left hand sides in relation to pathological diagnosis (p > 0.05).

Analysis by finger revealed that the third finger (n = 44; 32.8%) and first finger (n = 34; 25.4%) were the most commonly affected. However, there was no statistically significant difference in the incidence of tumor types among different fingers (p = 0.628) (Table 1).

Regarding pathological diagnoses, the most common lesion was TSGCT (37.3%; n = 50), followed by GC (14.2%; n = 19), epidermal/epidermoid cyst (9.0%; n = 12), fibroma (6.7%; n = 9), glomus tumor (5.2%; n = 7), and, less frequently, lipoma, hemangioma, chondroma, pyogenic granuloma, and other rare lesions.

In terms of gender distribution, 83% of epidermal cysts occurred in males, and this difference was statistically significant (p = 0.039). Although 71% of glomus tumors were found in female patients, significance could not be determined due to the small sample size (p > 0.05). Among TSGCT cases, 54% were male (n = 27) and 46% were female (n = 23), with no statistically significant difference observed (p > 0.05). GCs showed a female predominance (58%), but this difference was not statistically significant. Demographic data by tumor type are summarized in Table 2.

**Table 2:** Clinicopathological distribution according to tumor and tumor-like lesion types Data are presented as n (%) and mean values. GC, ganglion cyst; TSGCT, tendon sheath giant cell tumor

Diagnosis	n (%)	Age (mean, years)	Male, n (%)	Female, n (%)	Right, n (%)	Left, n (%)	Dorsal, n (%)	Volar, n (%)
TSGCT	50 (37.3%)	45.8	27 (54.0%)	23 (46.0%)	28 (56.0%)	22 (44.0%)	18 (36.0%)	32 (64.0%)
GC	19 (14.2%)	52.4	8 (42.1%)	11 (57.9%)	11 (57.9%)	8 (42.1%)	11 (57.9%)	8 (42.1%)
Epidermal cyst	12 (8.9%)	44.2	10 (83.3%)	2 (16.7%)	3 (25.0%)	9 (75.0%)	4 (33.4%)	8 (66.6%)
Fibroma	9 (6.7%)	46.3	2 (22.2%)	7 (77.8%)	6 (66.7%)	3 (33.3%)	4 (44.4%)	5 (55.6%)
Glomus tumor	7 (5.2%)	39	2 (28.6%)	5 (71.4%)	4 (57.1%)	3 (42.9%)	7 (100.0%)	0 (0.0%)
Foreign body granuloma	7 (5.2%)	48.6	4 (57.1%)	3 (42.9%)	3 (42.9%)	4 (57.1%)	3 (42.9%)	4 (57.1%)
Hemangioma	6 (4.5%)	48.7	2 (33.3%)	4 (66.7%)	1 (16.7%)	5 (83.3%)	1 (16.7%)	5 (83.3%)
Lipoma	4 (3.0%)	49.2	2 (50.0%)	2 (50.0%)	1 (25.0%)	3 (75.0%)	3 (75.0%)	1 (25.0%)
Soft tissue chondroma	4 (3.0%)	46.8	1 (25.0%)	3 (75.0%)	2 (50.0%)	2 (50.0%)	2 (50.0%)	2 (50.0%)
Pyogenic granuloma	3 (2.2%)	56	3 (100.0%)	0 (0.0%)	0 (0.0%)	3 (100.0%)	2 (66.7%)	1 (33.3%)
Neuroma	3 (2.2%)	40.3	1 (33.3%)	2 (66.7%)	1 (33.3%)	2 (66.7%)	1 (33.3%)	2 (66.7%)
Neurofibroma	2 (1.5%)	66	1 (50.0%)	1 (50.0%)	1 (50.0%)	1 (50.0%)	0 (0.0%)	2 (100.0%)
Angiolipoma	2 (1.5%)	51	0 (0.0%)	2 (100.0%)	0 (0.0%)	2 (100.0%)	0 (0.0%)	2 (100.0%)
Cavernous hemangioma	1 (0.7%)	62	0 (0.0%)	1 (100.0%)	0 (0.0%)	1 (100.0%)	0 (0.0%)	1 (100.0%)
Fibrolipoma	1 (0.7%)	28	1 (100.0%)	0 (0.0%)	1 (100.0%)	0 (0.0%)	0 (0.0%)	1 (100.0%)
Myopericytoma	1 (0.7%)	63	0 (0.0%)	1 (100.0%)	0 (0.0%)	1 (100.0%)	1 (100.0%)	0 (0.0%)
Schwannoma	1 (0.7%)	31	1 (100.0%)	0 (0.0%)	1 (100.0%)	0 (0.0%)	0 (0.0%)	1 (100.0%)
Tophus	1 (0.7%)	82	1 (100.0%)	0 (0.0%)	0 (0.0%)	1 (100.0%)	1 (100.0%)	0 (0.0%)
Hidrocystoma	1 (0.7%)	61	1 (100.0%)	0 (0.0%)	1 (100.0%)	0 (0.0%)	1 (100.0%)	0 (0.0%)

Comparison of clinical and pathological diagnoses revealed an overall concordance rate of 76.1% (p < 0.001). The initial diagnostic accuracy was high for TSGCT, GCs, and epidermal cysts. However, for fibroma cases, only 33.3% were correctly diagnosed preoperatively, while 66.7% were misdiagnosed. Similarly, the correct initial diagnosis rate was low for chondromas and other rare tumors. Based on these findings, fibroma was the most frequently misdiagnosed lesion. No significant difference was observed in mean age across different tumor types (p = 0.210) (Table 3).

**Table 3:** Comparison of pathological diagnoses with demographic and clinical variables Chi-square and one-way ANOVA tests were used. Statistical significance was set at p < 0.05.

Variable	Test	Test statistic	p-Value
Pathologic diagnosis - Preliminary diagnosis agreement	Chi-square	χ² = 34.7	0.001
Pathologic diagnosis - Gender	Chi-square	χ² = 4.26	0.039
Pathologic diagnosis - Age	ANOVA	F = 1.32	0.21
Pathologic diagnosis - Side (right/left)	Chi-square	χ² = 0.26	0.612
Pathologic diagnosis - Direction (dorsal/volar)	Chi-square	χ² = 7.21	0.027

## Discussion

The vast majority of masses in the hand and fingers are benign and are surgically treated due to functional impairment or cosmetic concerns. In this study, we focused exclusively on masses located in the fingers and found that TSGCT was the most common pathological diagnosis among 134 cases. While TSGCT is the most frequent cause of hand tumors overall, ganglion cysts at the finger level were relatively less common, consistent with the literature [8]. Tang et al. [9] reported that the rate of TSGCT in masses distal to the metacarpophalangeal joint is higher than that of ganglia, and in our series, the rate of TSGCT (37%) exceeded this value. In contrast, ganglia, which typically constitute 50-70% of general hand masses, accounted for only 14% in our finger series [8].

TSGCTs are generally observed in patients aged 30-50, with a slight female predominance [8]. The mean age in our series was approximately 46 years, consistent with existing reports. Although the female-to-male ratio is generally balanced (~3:2 or 1:1), some studies, such as Uslu et al., report a significant female predominance (79% in a series of 95 cases) [10]. In our study, no significant gender difference was observed (46% female). TSGCTs typically occur around the flexor tendon sheaths, and in our series, two-thirds of cases were volar, consistent with the literature.

Dorsal wrist ganglia constitute approximately 70% of all ganglia, with a smaller proportion on the volar wrist and fingers. In our series, ganglia were the second most common lesion, with 58% of patients being female, reflecting the known female predominance [8]. While ganglia can occur at any age, they are more frequent in middle-aged adults. Clinical diagnosis is generally straightforward; in our series, the initial diagnostic accuracy was 89%.

Epidermal inclusion cysts (EICs) typically develop on the hand after trauma or surgery, resulting in subcutaneous implantation of dermal epithelium. They are reported as the third most common benign hand mass [11]. Lincoski et al. reported that 16% of 623 excised hand masses over 27 years were epidermoid cysts [12]. In our series, 10 of 12 EIC patients (83%) were male, consistent with literature suggesting a higher prevalence in male workers exposed to repetitive minor trauma [11]. Most EICs occur as solitary lesions on the volar surface of distal fingers; in our series, 67% were volar, and most had a history of trauma. The high frequency of manual labor in this industrial region likely contributed to the increased prevalence of TSGCT and EIC cases.

Glomus tumors are small, extremely painful lesions, usually located on the fingertips or subungual region. They account for approximately 1% of hand tumors and are often undiagnosed for years. These tumors are more common in adults aged 20-50 and predominantly affect women [13]. Literature reports a recurrence rate of 5-10%, which increases after multiple surgeries. Our seven glomus cases (5.2%) fall within the reported range. Seventy-one percent of these patients were female, with a mean age of approximately 39 years. All lesions were subungual, consistent with prior reports.

Soft tissue chondromas are benign cartilage tumors not attached to bone, most commonly occurring in fingers and toes, though the overall incidence is very low [14]. They are thought to be associated with recurrent microtrauma [15]. Four cases (3%) in our series likely reflect the predominance of industrial workers and farmers in our patient population, supporting the microtrauma hypothesis. Clinically, these tumors present as firm, slow-growing, painless nodules. Diagnosis is confirmed histopathologically; our cases were reported as “benign chondromatous lesion containing hyaline cartilage islands.” Marginal excision is the treatment of choice, with low recurrence if completely removed. Literature reports a 15-20% recurrence rate with incomplete excision and rare malignant potential [14,16]. Our cases ranged from 31 to 62 years, with three females. Misdiagnosis as fibroma likely reflects the rarity and macroscopic similarity of these tumors to fibroma.

Tendon sheath fibroma is another rare benign soft tissue tumor of the hand, composed of fibroblast and myofibroblast proliferation, forming firm nodules attached to flexor tendons [17]. In a large series by Chung and Enzinger (138 cases), 82% of lesions were in the hand/fingers, mostly on the flexor side [18]. Histologically, these tumors are lobulated with dense collagenous stroma. Recent molecular studies identified USP6 gene fusion in some subtypes, suggesting a clonal neoplasm similar to nodular fasciitis [19]. Fibroma can be clinically confused with TSGCT; most of our cases were initially misdiagnosed as giant cell tumors. Literature suggests a slightly higher prevalence in men [17,20]; in contrast, most of our nine cases were women.

Limitations

One limitation of this study is that not all patients with soft tissue masses of the fingers present to our institutions, which limits the accuracy of regional incidence estimates. Multicenter studies would therefore provide more robust data. Additionally, long-term oncological outcomes were not the primary focus of this retrospective analysis, and this has been explicitly acknowledged as a limitation. Given the retrospective and exploratory nature of the study, no prior sample size or power calculations were performed. Standard assumptions for the statistical tests applied were evaluated, with a p-value < 0.05 considered statistically significant. Multiple comparison correction was not applied, which is another methodological limitation.

## Conclusions

Overall, our results are largely consistent with the literature, though certain discrepancies were noted. TSGCT was the most common lesion among benign tumors of the fingers, GCs were less frequent than reported elsewhere, and fibromas were more prevalent in women. These differences may relate to the high level of manual labor in this industrial population, resulting in increased exposure to repetitive microtrauma of the hands. Some lesions, such as epidermal cysts and glomus tumors, demonstrated male or female predominance, which was also observed in our series.

The overall clinical prediagnostic success was high (76%), though accuracy decreased for rare tumors, highlighting that preoperative diagnosis can be challenging even for experienced teams. Differential diagnosis is particularly difficult for lesions that closely resemble TSGCT, including fibromas and chondromas. Although benign, some tumors, particularly TSGCT and glomus tumors, carry a risk of recurrence, emphasizing the importance of complete surgical excision. A high index of suspicion should be maintained for small but painful lesions such as glomus tumors, especially in cases of recurrent pain. Because soft tissue tumors of the fingers encompass a wide spectrum, thorough clinicoradiologic evaluation is essential to support histopathologic diagnosis. Knowledge of the incidence and typical features reported in the literature can facilitate accurate diagnosis and guide appropriate curative treatment. The data from this study provide valuable insights to assist clinicians in the diagnosis and management of finger masses.
